# Cognitive Behavioral Performance of Untreated Depressed Patients with Mild Depressive Symptoms

**DOI:** 10.1371/journal.pone.0146356

**Published:** 2016-01-05

**Authors:** Mi Li, Ning Zhong, Shengfu Lu, Gang Wang, Lei Feng, Bin Hu

**Affiliations:** 1 International WIC Institute, Beijing University of Technology, Beijing, China; 2 Beijing International Collaboration Base on Brain Informatics and Wisdom Services, Beijing, China; 3 Beijing Key Laboratory of MRI and Brain Informatics, Beijing, China; 4 Maebashi Institute of Technology, Maebashi, Japan; 5 Mood Disorders Center & China Clinical Research Center for Mental Disorders, Beijing Anding Hospital, Capital Medical University, Beijing, China; 6 Center of Depression, Beijing Institute for Brain Disorders, Beijing, China; 7 Ubiquitous Awareness and Intelligent Solutions Lab, Lanzhou University, Lanzhou, China; Istituto Superiore di Sanità, ITALY

## Abstract

This study evaluated the working memory performance of 18 patients experiencing their first onset of mild depression without treatment and 18 healthy matched controls. The results demonstrated that working memory impairment in patients with mild depression occurred when memorizing the position of a picture but not when memorizing the pictures themselves. There was no significant difference between the two groups in the emotional impact on the working memory, indicating that the attenuation of spatial working memory was not affected by negative emotion; however, cognitive control selectively affected spatial working memory. In addition, the accuracy of spatial working memory in the depressed patients was not significantly reduced, but the reaction time was significantly extended compared with the healthy controls. This finding indicated that there was no damage to memory encoding and function maintenance in the patients but rather only impaired memory retrieval, suggesting that the extent of damage to the working memory system and cognitive control abilities was associated with the corresponding depressive symptoms. The development of mild to severe depressive symptoms may be accompanied by spatial working memory damage from the impaired memory retrieval function extending to memory encoding and memory retention impairments. In addition, the impaired cognitive control began with an inadequate capacity to automatically process internal negative emotions and further extended to impairment of the ability to regulate and suppress external emotions. The results of the mood-congruent study showed that the memory of patients with mild symptoms of depression was associated with a mood-congruent memory effect, demonstrating that mood-congruent memory was a typical feature of depression, regardless of the severity of depression. This study provided important information for understanding the development of cognitive dysfunction.

## Introduction

Numerous studies have confirmed that patients with depression have emotional and cognitive deficits [1–4], with executive, language, and working memory dysfunctions [5–9]. After completing memory tasks, patients with depression performed significantly worse than the normal population [5]. Working memory [10] provides temporary storage and a limited processing system for cognitive information. It comprises the cognitive bases of verbal understanding, reasoning, problem solving, and learning. Working memory is divided into spatial working memory and non-spatial working memory (including words and objects) based on memory objects, with spatial working memory more active in the right frontal hemisphere and non-spatial working memory more active in the left frontal hemisphere [11]. A study has shown that the right hemisphere has more conductive to spatial working memory, and the left hemisphere is more conductive to non-spatial working memory [12]. Previous studies of working memory have primarily involved research on the executive function, cognitive control function, non-spatial working memory of words or objects, spatial working memory, relationship between emotional and spatial working memory, and mood-congruent memory. An imaging study has demonstrated abnormal executive function and a template of visuospatial function in the working memory of patients with depression [13]. Studies have also demonstrated that the impaired working memory in patients with depression primarily showed deficits in executive function [14, 15]. The study using the Sternberg’s working memory paradigm showed that executive dysfunction in the prefrontal cortex resulted in a significant decline in working memory capacity in patients with untreated severe depression [14]. These patients demonstrated a high error rate in the working memory task and an extended response time [16]. Schatzberg et al. have observed significant impairments in attention and verbal memory in patients with depression [17]. A word memory study by Fossati et al. showed that patients with depression performed normally in a cued-recall and a recognition memory task, but were impaired in free recall [18]. Harvey et al. have used an n-back working memory paradigm for verbal memory study. The accuracy of the working memory of patients with severe depression was lower than that of healthy controls, and the response time of patients with severe depression was significantly longer than that of healthy controls [2]. Rose and Ebmeier used a symbolic n-back experimental paradigm and showed lower memory accuracy and a longer response time in patients with depression compared with healthy controls [19]. Some studies have suggested that the working memory of patients with depression declines as the task difficulty increased [2, 3, 6].

Many studies have focused on the relationship between emotion and cognition. Research has shown that negative emotion has different impacts on the words and spatial working memory in normal subjects [20, 21]. Lavric et al. used a word n-back working memory task and spatial n-back working memory task and showed that negative emotion had no impact on the results of the word memory task but did affect spatial working memory [20]. Li et al. have also demonstrated that negative emotion did not impact word memory tasks but did impact spatial working memory, with a decline in spatial working memory retention capacity [22]. These results indicated that negative emotion selectively affects spatial working memory. This type of impact was explained as follows: Shackman believed that negative emotion and spatial working memory both have advantages in the right hemisphere of the brain. This overlap of cortical functions allows them to mutually affect each other [21]. Weiland-Fiedler et al. believed that depression primarily damaged the right hemisphere of the brain, resulting in more influences on the right hemisphere, which is associated with spatial working memory [1]. Lavric et al. believed that the emotional impact of memory might not result in competition in the cortical region but rather a competition of resources. Spatial working memory required more attention resources compared with word memory, thereby resulting in functional damage to spatial working memory [20]. Hertel et al. have suggested that patients with depression experience a loss of cognitive control function, leading to decreased working memory capacity [23]. However, some studies have observed damage to both verbal and spatial working memory in patients with depression [24]. The differences among these studies may be caused by different memory material and the depression severity in the tested subjects.

Studies of mood-congruent memory [25, 26] have suggested that stimuli that are consistent with emotion are more easily remembered [27], that is, people with negative emotion could easily remember the negative information [28]. Storbeck and Clore induced different emotions in the subjects to study mood-congruent memory and found that subjects with an induced negative emotional status had a more accurate memory than subjects with an induced positive emotional status [29]. Patients with depression tended to remember more negative stimuli that were consistent with their mood compared with the healthy controls. In contrast, these patients rarely remembered the positive stimuli that were inconsistent with their mood [30]. The enhanced memory of mood-congruent negative stimuli and the reduced memory of positive stimuli that were inconsistent with the mood might represent the cognitive control ability of patients with depression [31, 32].

Although the results of studies on mood-congruent memory and on the selective influence of negative emotion appear inconsistent, they are not contradictory. Negative emotions selectively affect spatial working memory, which explains the differences in memory accuracy and response times (inter-group comparisons) associated with different emotions between the patient and healthy control groups. Mood-congruent memory studies have observed differences in the memory accuracy of subjects experiencing negative or positive emotional states (in intra-group comparisons). Hence, using different perspectives, these two study types reveal memory effects among depressed patients.

Although many studies have observed working memory damage in patients with depression, regardless of whether they are simple working memory studies in patients with depression or studies of the correlation between emotion and memory, there have been inconsistent findings and conclusions. There are several possible explanations for these discrepancies. (1) Memory material differences affect memory performance. For example, word memory encoding is localized in the left prefrontal cortex, while the encoding of facial emotional memory involves the bilateral prefrontal cortex [33]. (2) The differences in the display time of memory material affect memory performance. For patients with depression and attention deficit, the short display time of memory stimuli affects the memory encoding. This memory impairment may not be associated with a difference in the memory system but may mostly reflect the lack of attention resources [20]. (3) Most studies have focused on patients with severe depression. However, the severity of depression (e.g., mild and moderate) and the different courses of the disease (e.g., working memory) are major factors that affect the cognitive performance [6, 14, 16]. (4) Studies of spatial working memory impairment in patients with depression originate from the use of task paradigms of spatial working memory comparing patients with depression to healthy controls. There is insufficient comparative analysis evidence for both spatial working memory and non-spatial working memory between patients with depression and healthy controls. Otherwise, most previous studies have focused on the working memory of patients with severe depression but have rarely reported on patients with mild depression who can live and work normally [22, 24].

In this study, we assessed patients with a first onset of untreated mild depression. We used positive, neutral, and negative emotional pictures that were based on the modified Sternberg’s working memory paradigm to evaluate the working memory in terms of pictures and picture position, cognitive control, and mood-congruent memory in patients with depression and healthy controls.

## Materials and Methods

### Ethics statement

All subjects provided signed informed consent and this study was approved by the Ethics committee at Beijing Anding Hospital, Capital Medical University, China.

### Participants

Thirty-six subjects participated in this study, including 18 patients with depression (i.e., first onset and untreated mild depression) and 18 matched (according to age, gender and education level of depressed patients) healthy volunteers. The patients were recruited at their first visit to the outpatient clinic at Beijing Anding Hospital, Capital Medical University, Beijing, China, and the healthy volunteers were recruited through promotional posters. All patients were diagnosed with depression according to the Diagnostic and Statistical Manual of Mental Disorders, Fourth Edition (DSM-IV). Severity of depression was assessed using Clinical Global Impression- severity of illness (CGI-SI), in which the CGI-SI was scored by one doctor, and can divide the severity of depression into eight level (a score from 0–7): 3 is mild and 4 is moderate. In addition, the 17 items of the Hamilton depression rating scale (HAMD) scored by another doctor, and the Quick inventory of depressive symptomatology-self report (QIDS-SR_16_) were used to assess the depression. Only when the results assessed by the CGI-SI, HAMD and QIDS-SR_16_ are consistent (mild or moderate), the patients could be enrolled. The following inclusion criteria were used for the patient group: (1) age ranging from 18 to 60 years, right-handed, fulfilled the diagnostic criteria for depression according to the DSM-IV; (2) diagnosed with significant, mild to moderate depression and capable of working and living normally (CGI = 3 or 4); (3) severity of depression was assessed using the HAMD with 7 < HAMD < 24; (4) self-assessment based on the QIDS-SR_16_ with 5 < QIDS-SR_16_ < 18; (5) experiencing the first onset of depression without receiving any treatment or antidepressant drugs; (6) no colorblindness or other eye diseases, with normal vision or normal corrected vision, and able to complete an eye movement experiment. The inclusion criteria for the healthy volunteers were as follows: (1) age ranging from 18 to 60 years, right-handed; (2) self-assessment based on the QIDS-SR_16<_ 5; Beck depression inventory (BDI) < 4; (3) no administration of any psychotropic drugs intervening with the function of nervous system, no previous history of depression and mental illness, no alcohol or other dependence. Subjects were given the payment after completion of the experiment.

### Experimental materials

This study used three types of experimental pictures, including 60 samples each of positive, neutral, and negative pictures. All pictures were obtained from the International Affective Picture System (IAPS), with an average valence of 7.31 ± 0.44 and an average arousal of 5.54 ± 0.44 for the positive pictures, an average valence of 2.79 ± 0.51 and an average arousal of 5.97 ± 0.44 for the negative pictures, and an average valence of 5.18 ± 0.17 and an average arousal of 3.23 ± 0.22 for the neutral pictures. After picture processing using Picture Manager Software (Adobe Photoshop 6.0), the size, grayscale, and resolution were identical in all images.

### Experimental paradigm and procedures

This study used a modified Sternberg working memory (WM) paradigm [34]. To examine the consistency of mood and memory, each WM encoding task prompted four pictures depicting a specific type of emotions (i.e., four positive, four negative, or four neutral pictures). These four pictures corresponded to four different positions (upper left, upper right, lower left, and lower right). The WM encoding task used in the experiment included positive, negative, and neutral pictures. The probe stimulus, and WM encoding task picture types were identical. Probe stimuli were divided into two types: a picture probe presenting at the center of the screen and a picture position probe presenting at any location.

**Fig 1:**
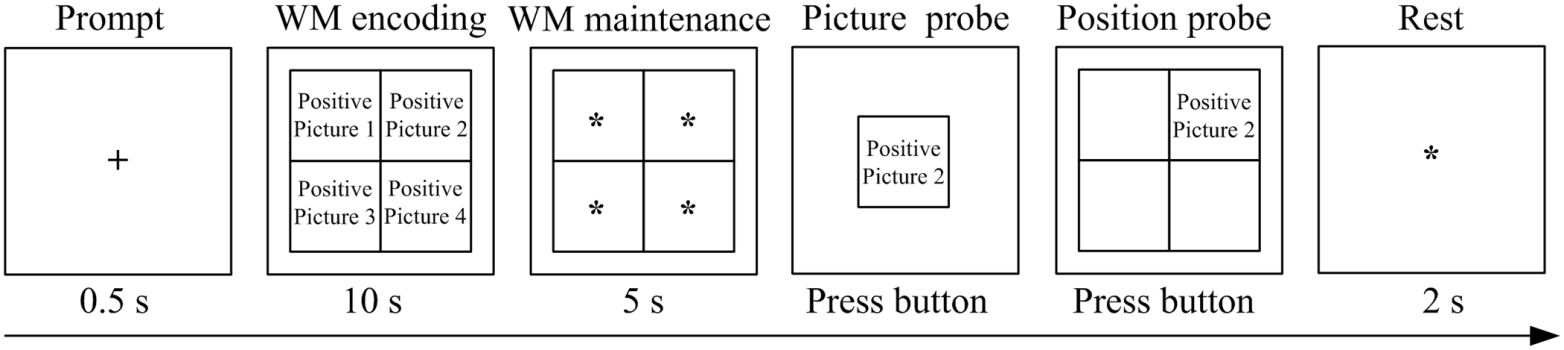
A schematic representation of an example trial of the working memory (WM) task in our experiment. Each trial included a symbol of “+” as a prompt (0.5 seconds); and a WM encoding task including the same emotional type of four pictures (10 seconds), followed by a WM maintenance task (5 seconds); and then a picture probe and a position probe to ask the subjects to judge by pressing the buttons, followed by a 2-second rest.

Experimental procedure: As shown in Fig 1, first, a symbol of “+” was shown as a prompt (0.5 seconds) at the center of the screen, suggesting to the subjects that the WM task would immediately appear. Then, the WM encoding task was presented for 10 seconds. The subjects were instructed to memorize each picture and its corresponding position during 10 second periods. After the encoding task disappeared, a five-second WM maintenance was given; then, a probe picture was shown at the center of the screen, and the subjects were asked to judge whether the probe picture was previously shown. Subjects were required to press the left button if the probe had been shown previously and the right button if the probe had not been previously shown. After the subjects pressed the button, the same probe picture was shown (the picture position probe) and the subject was asked if the picture had been shown at this particular position. The subjects were required to press the left button if their answer was “yes” and the right button if their answer was “no”. If the subjects selected the right button for the judgment of the probe picture, the picture position assessment would be ignored, and a symbol of “*” asterisk would be presented, suggesting a break for two seconds, followed by presentation of the next trial.

### Statistical analysis

SPSS 20.0 software (SPSS, Chicago, IL, USA) was used for the statistical analyses. We employed a mixed-model analysis of variance (ANOVA) with 2 (group: depressed, control) × 3 (within-subject factor stimulus material: positive, neutral, negative) repeated measures analysis on the accuracy and response time of the picture and position working memory, and we used the Bootstrapped two-way ANOVA (1000 bootstrap samples) to verify; we included gender, age and education level as covariates. Comparisons of between-group in age and years of education were performed using an independent-samples t-test; pair-wise inter-group comparisons were performed using independent-samples t-tests, and the Bootstrapped independent-samples t-tests (1000 bootstrap samples) were used to verify; pair-wise intra-group comparisons on the three emotional picture types were performed using paired-samples t-tests, the Bonferroni method was applied to conduct multiple comparison corrections, and we also used the Bootstrapped paired-samples t-tests (1000 bootstrap samples) analysis. The comparison of gender distribution between the groups (depressed patients and healthy volunteers) was performed using a chi-squared test. In addition, we also analyzed the effect size. To analyze variance, the effect size was computed to obtain *ω*^2^ (eta squared) value. According to the literature [35], the evaluation criteria have small (.01 ≤ *ω*^2^ < .06), moderate (.06 ≤ *ω*^2^ < .14), and large effects (*ω*^2^ ≥ .14). For independent samples and paired samples t-tests, we calculate Cohen’s d value, which is the most commonly standardized effect size. The Cohen evaluation criteria [36] have small (.2 ≤ *d* < .5), moderate (.5 ≤ *d* < .8), and large effects (*d* ≥ .8).

## Experimental results and analysis

### Demographic and clinical data analyses

The demographics and clinical data were shown in Table 1.

**Table 1:** Demographic and clinical data.

Variables (*Mean* ± *S*.*D*.)	Depressed patients (n = 18)	Healthy controls (n = 18)	p-value
Gender (males: females)	8: 10	9: 9	.738
Age (years)	34.28 ± 10.12	33.56 ± 8.48	.818
Education level (yeas)	13.61 ± 3.52	14.50 ± 3.45	.449
QIDS-*SR*_16_	16.56 ± 3.35	2.50 ± 1.58	.000
HAMD (17-item)	16.06 ± 4.35		

*QIDS*—*SR*_16_, quick inventory of depressive symptomatology-self report; HAMD, the Hamilton depression rating scale.

### Comparing the between-group picture working memory accuracy and response time

**Fig 2:**
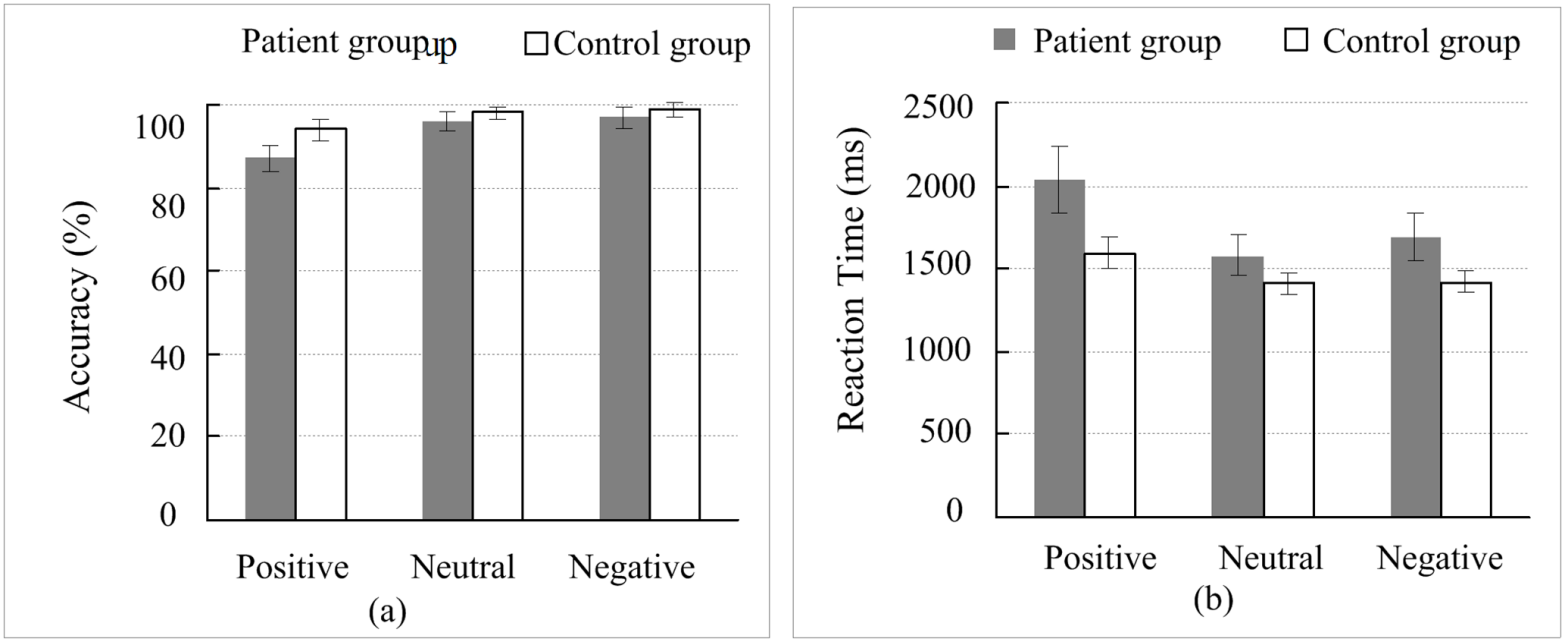
Comparison of the accuracy and response time of picture working memory between groups. (a) The accuracy of picture working memory; (b) the response time of picture working memory. Error bars represent the standard error of the mean (SEM).

As shown in Fig 2, the patients had lower memory accuracy for all emotional types of pictures (i.e., positive, neutral, and negative) compared with the healthy controls, and the patient response time for picture recognition was longer than that of the healthy controls. Here, we performed a statistical analysis of the between-group differences.

The accuracy of picture working memory: a mixed-model ANOVA with 2 (depressed, control) × 3 (positive, neutral, negative) repeated measures analysis showed that the group factor had no significant effect on the picture working memory [*F*(1, 31) = 2.723, *p* = .109, *ω*^2^ = .043], which was consistent with the result based on the Bootstrapped two-way ANOVA (*p* = .079). The response time of picture working memory: a mixed-model ANOVA with 2 (depressed, control) × 3 (positive, neutral, negative) repeated measures analysis showed that the group factor had no significant effect on the picture working memory [*F*(1, 31) = 2.625, *p* = .115, *ω*^2^ = .041], and the Bootstrapped two-way ANOVA analysis showed the consistent results (*p* = .058).

### Comparing the accuracy and response time of position working memory between the groups

**Fig 3:**
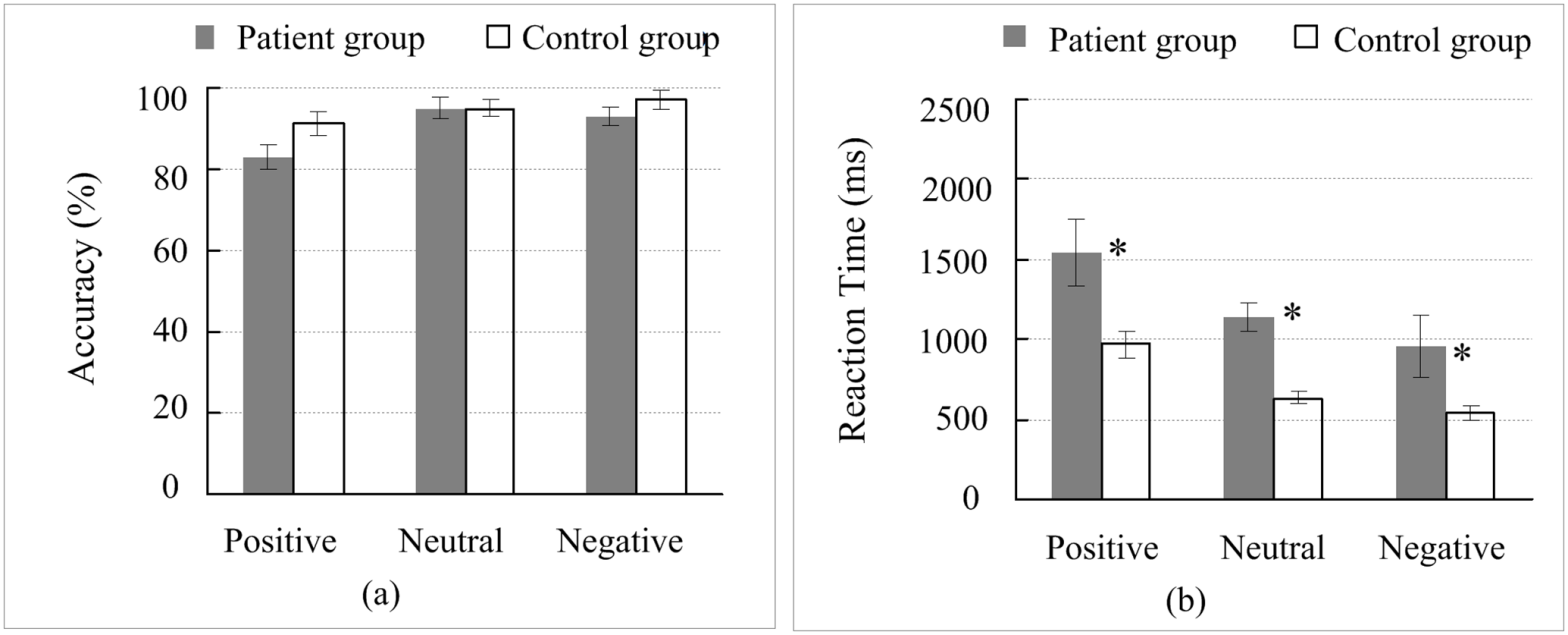
Comparison of the accuracy and response time of working memory for picture position between the groups. (a) The accuracy of picture position working memory; (b) the response time of picture position working memory. *, *p* < 0.05; Error bars represent the standard error of the mean (SEM).

As shown in Fig 3, the patients demonstrated lower accuracy in position working memory for all picture types (i.e., positive, neutral, and negative) compared with the healthy controls, and the patient response time for position recognition was also longer than that of the healthy controls. Here, we performed a statistical analysis of the between-group differences.

The accuracy of position working memory: a mixed-model ANOVA with 2 (depressed, control) × 3 (positive, neutral, negative) repeated measures analysis showed that the group factor had no significant effect on the position working memory [*F*(1, 31) = 2.308, *p* = .139, *ω*^2^ = .036], which was consistent with the result based on the Bootstrapped two-way ANOVA (*p* = .064). The response time of position working memory: a mixed-model ANOVA with 2 (depressed, control) × 3 (positive, neutral, negative) repeated measures analysis showed that the group factor had significant effect on the position working memory [*F*(1, 31) = 14.557, *p* = .001, *ω*^2^ = 0.257], which was also consistent with the result based on the Bootstrapped two-way ANOVA (*p* = .001). To further analyze the between-group differences, we performed an independent-sample t-test on the response time of the position in positive, neutral and negative pictures between the groups. The results showed that all of the between-group differences for the three types of pictures were significant [positive: *t*(21.348) = 2.546, *p* = .019, *d* = .873, Bootstrapped *p* = .048; negative: *t*(22.159) = 4.625, *p* = .000, *d* = 1.585, Bootstrapped *p* = .001; neutral: *t*(18.756) = 2.680, *p* = .015, *d* = .920, Bootstrapped *p* = .023].

### Analysis of mood-congruent memory

**Fig 4:**
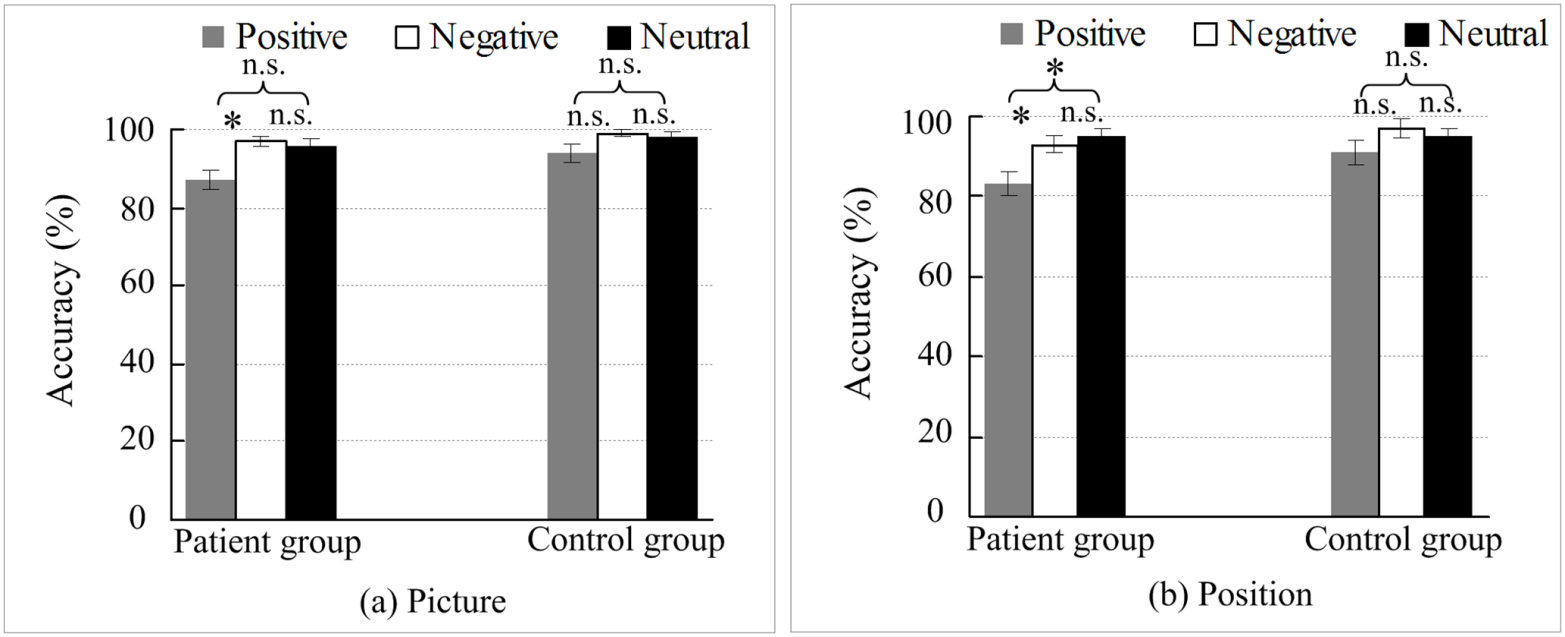
Analysis of mood-congruent memory. (a) Analysis of picture working memory of mood-congruent memory; (b) analysis of picture position working memory of mood-congruent memory. *, P < 0.05; Error bars represent the standard error of the mean (SEM); n.s. represents a non-significant difference.

A meta-analysis showed that compared with healthy volunteers, patients with depression tend to remember more negative stimuli that are consistent with their negative moods and remember less positive stimuli that are inconsistent with their moods [30]. To further analyze the differences in emotional picture types, pair-wise intra-group comparisons on the accuracy of three emotional picture types were performed using paired-samples t-tests, and the Bonferroni method was applied to conduct multiple comparison corrections. In addition, we also used the Bootstrapped paired-samples t-tests to verify the results. For the accuracy of picture working memory, the results of 2 (depressed, control) × 3 (positive, neutral, negative) repeated measures ANOVA showed that the Mauchly’s test of sphericity on within-subject factor (emotion) is: Mauchly *W* = .982, approximately *chi* − *square* = .534, *p* = .766 > .05. For the accuracy of position working memory, the results of 2 (depressed, control) × 3 (positive, neutral, negative) repeated measures ANOVA showed that the Mauchly’s test of sphericity on within-subject factor (emotion) is: Mauchly *W* = .989, approximately *chi* − *square* = .346, *p* = .841 > .05. The accuracy of the picture and position working memory are shown in Fig 4.

Fig 4(a) shows that the memory accuracy of the negative pictures in the patients was significantly higher than that of the positive pictures [*t*(17) = 2.824, *p* = .036, *d* = .920], which was consistent with the result based on Bootstrapped paired t-test (*p* = .019); the memory accuracy of the negative and positive pictures in the healthy controls had no significant difference [*t*(17) = 1.117, *p* = .837, *d* = .510], which suggest that picture memory is associated with a mood-congruent effect. As shown in Fig 4(b), the memory accuracy of the position of the negative pictures in the patients was significantly greater than the memory accuracy of the position of the positive pictures [*t*(17) = 2.882, *p* = .030, *d* = .822; Bootstrapped *p* = .021]. The memory accuracy of the position of the negative and positive pictures in the healthy controls had no significant difference [*t*(17) = 1.954, *p* = .201, *d* = .368; Bootstrapped *p* = .078], which suggest that the memory of picture position is associated with a mood-congruent effect. Fig 4(a) shows that there was no significant difference between the memory accuracy of the neutral pictures and the positive pictures in the patients [*t*(17) = 2.339, *p* = .096, *d* = 0.769]. Although the results of the multiple comparison correction did not reach the level of significance, the effect size (Cohen’s d) was close to the large effect, thus the difference in accuracy between positive pictures and neutral pictures was close to significant. Actually, the Bootstrapped result showed that the neutral pictures in the accuracy was significantly higher than the positive pictures (*p* = .036), which verifies that the Cohen’s d is unrelated to the sample size. The accuracy of the position of the neutral pictures in the patients was significantly higher than that of the positive pictures [*t*(17) = 2.798, *p* = .036, *d* = 1.104; Bootstrapped *p* = .014]. In the healthy controls, no significant differences were found between the positive and negative picture types [*t*(17) = 1.166, *p* = .780, *d* = .205; Bootstrapped *p* = .327] and picture positions [*t*(17) = 1.930, *p* = .210, *d* = .552; Bootstrapped *p* = .081]. Additionally, Fig 4 also shows that in patients with depression and healthy controls, no significant difference was found between the memory accuracy of the neutral pictures and negative pictures [patients: *t*(17) = 0.448, *p* = 1.000, *d* = 0.102; Bootstrapped *p* = .717; controls: *t*(17) = .137, *p* = 1.000, *d* = .146; Bootstrapped *p* = .801]; and no significant difference was found between the accuracy of the position of the neutral pictures and that of the negative pictures [patients: *t*(17) = .446, *p* = 1.000, *d* = .194; Bootstrapped *p* = .679; controls: *t*(17) = .903, *p* = 1.000, *d* = .229; Bootstrapped *p* = .371].

## Discussion

This study investigated the working memory and mood-congruent memory of patients with first onset untreated mild depression. No significant difference in the picture types (i.e., positive, negative, and neutral) working memory capacity (accuracy and response time) was found between the patients with depression and the healthy controls (Fig 2). The results indicated that the object (picture) working memory capacity of the patients with first onset untreated mild depression was lower than that of the healthy controls to some extent, but the difference did not reach the level of significance. However, patients with depression showed significantly decreased in the response time of the position working memory of the picture (i.e., positive, negative, and neutral) compared with the healthy controls (Fig 3), indicating that the working memory impairment of patients with depression was demonstrated as spatial working memory impairment. This result was consistent with the results of other studies [20, 21]. Previous studies have demonstrated that working memory impairment showed not only a decline in memory accuracy but also a significant extension of response time [2, 20, 21, 24]. Even for patients with untreated severe depression, their working memory impairment presented as a decline in memory accuracy and significantly longer response time [14]. In contrast to the previous findings, this study did not find a significant decline of memory accuracy in the patients with depression but observed a significant extension of response time compared with the healthy controls, indicating that there was no functional impairment of memory encoding in patients; however, there was functional impairment of memory retrieval. These findings may imply that the severity of impairment in the working memory system corresponded to the symptoms of depression. During the development of depressive symptoms from mild to severe stages, the working memory impairment began with functional impairment of memory retrieval and extended comprehensive damage to memory retrieval, memory encoding, and memory retention.

A previous study has shown that negative emotion selectively affected spatial working memory [20–22]. Although the results of this study were consistent with previous reports, including the impairment of spatial working memory (i.e., the picture position working memory) and no impairment of non-spatial working memory (i.e., the picture working memory), the memory accuracy and the response time showed that the impact of negative emotion (negative picture) on working memory was similar to the impact of positive emotion (positive picture) or non-emotion (neutral picture). No specific impact of external negative emotion (negative stimuli from the negative picture) on spatial working memory was observed. This finding suggested that of the impaired spatial working memory was not caused by the external negative emotion. Cognitive control might selectively affect spatial working memory, but the negative emotions did not selectively affect spatial working memory. Functional impairment of cognitive control led to a decreased working memory capacity in patients with depression [23]. Cognitive control function was changed even in patients with mild depression symptoms [37]. This type of change was also observed as an impact of selective cognitive control on the memory retrieval function of spatial working memory, but no significant impact on the encoding function of spatial working memory was found in this study. Previous studies have shown that the posterior parietal cortex is associated with attention, episodic memory and working memory [38, 39]. The posterior parietal cortex includes the dorsal parietal cortex (DPC) and ventral parietal cortex (VPC) [40]. Evidence suggests that DPC allocates effortful top-down attention for memory retrieval during cued retrieval, whereas VPC mediates spontaneous bottom-up memory retrieval during un-cued retrieval; in addition, activity in the DPC network has been associated with faster cued retrieval response times [41]. In the cued working memory test conducted in the present study, impaired DPC functions and functional connectivity disorders between DPC and MTL (medial temporal lobe) likely resulted in an inadequate top-down allocation of attention resources [41], thereby contributing to significantly prolonged (memory retrieval time) spatial working memory response times among the patients. However, depressed patients typically present with dysthymia symptoms due to their automatic retrieval and processing of unconscious negative events, which involves a bottom-up, un-cued memory retrieval process of the ventral parietal cortex (VPC). Although cued and non-cued memory retrieval involves different posterior parietal cortexes, cognitive controls cannot reasonably allocate time resources, leaving inadequate attention resources for cued memory retrieval. Consequently, patient spatial working memory response times (memory retrieval time) are significantly delayed. Note that no significant cognitive difference in memory retrieval function was observed between the negative emotional pictures and positive emotional/neutral emotional pictures (neutral pictures). These findings indicate that external positive and negative emotional information had the same effect on memory retrieval, indicating that inhibitory defects of internal unconscious negative emotional procession exist rather than reduced inhibitory capacity of external negative emotion for patients with mild depressive symptoms [42–44]. In other words, patients with mild depressive symptoms demonstrated no deficiency in the cognitive control of external emotion but were deficient in the cognitive control to suppress the automatic processing of internal negative emotion during the memory retrieval of the picture position. This cognitive control impairment led to the difficulties in memory retrieval. Automatic processing of internal negative emotion is the main cause of hypothymergasia and insufficiency of attentional resources. This finding may indicate a correlation between cognitive control ability and depressive symptoms. During the development of depressive symptoms from mild to severe stages, these symptoms may be accompanied by cognitive control function from a reduced inhibitory capacity of unconscious negative emotional processing extending to the inhibitory impairment of external emotion.

Our study of mood-congruent memory demonstrated the memory accuracy of the negative pictures and position of the negative pictures in the patients with depression was significantly greater than that of the positive picture and position of the positive pictures, whereas no significant difference was found between the two types of pictures and their positions in the healthy controls. For the neutral pictures, the subjects encoded the memory based on their emotional statuses (i.e., healthy controls encoded the neutral pictures with a positive mood, while the patients encoded it with a negative mood). Thus, patients with depression remembered more of the neutral pictures. From the perspective of attentional resource competition, the neutral pictures are non-emotional; their encoding process was not affected by emotional stimuli compared with the encoding of the positive pictures, and the attentional resources were relatively sufficient for patients with depression to pay more attention to the neutral pictures. In summary, although no significant difference was found the memory accuracy of the picture and picture position between patients with depression and healthy controls, patients with depression showed mood-congruent memory for both the picture and picture position. This finding suggests that patients with depression tended to remember the negative pictures and the position of the negative pictures rather than the positive pictures and the position of the positive pictures, in which is associated with a mood-congruent memory effect [30–32]. These findings may indicate that there is no correlation between mood-congruent memory and depression severity. Mood-congruent memory is a typical feature of patients with depression.

## Conclusions

This study demonstrated that patients with first onset untreated mild depression symptoms had selective cognitive control impacts; however, no impact was observed from negative emotion on spatial working memory. In addition, this impact applied to the response time, not memory accuracy. In other words, no impairment of spatial memory encoding and memory retention capacity was found in the patients with depression; the only impairment observed was on memory retrieval function. The results of this study, together with previous findings, suggest that depressive symptoms are associated with working memory performance and cognitive control capabilities. During the development of depression symptoms from the mild to severe stages, there will be an accompanying comprehensive decline in working memory capacity (from memory retrieval to memory encoding) and comprehensive impairment of cognitive control capacity (from internal suppression to external suppression of negative emotion). Although emotional stimuli (positive or negative) had nearly identical impact on the working memory of patients with depression and the healthy controls, the memory of patients with mild depressive symptoms was associated with a mood-congruent memory effect, suggesting that mood-congruent memory is a typical feature of depression that is not associated with the severity of depression. Further study with a larger sample size and more experiments will be necessary to confirm that mood-congruent memory is an internal behavioral index.
